# Bœnninghausen’s Aphorisms of Hippocrates

**Published:** 1887-05

**Authors:** 


### BŒNNINGHAUSEN'S APHORISMS OF HIPPOCRATES.

TRANSLATED BY A. MCNEIL, M. D., SAN FRANCISCO, CAL.

### APHORISM 65, BOOK VII.

He who gives to a fever patient the nourishment suitable for the healthy should remember that that which strengthens the convalescent will make the sick still worse.

This aphorism belongs, without doubt, to the truest and most important maxims of the entire collection, and corresponds as well to the laws of nature as to those of every rational, medical science.

In the glossary to *Aphorism* 59, Book VII, we have had occasion to touch this subject, but only on one side, while this is on the other, then of fasting and hunger, and particularly where they were not indicated by nature. Here it relates to the blameworthy custom of not only relatives and nurses, but even occasionally of ignorant physicians, who press patients to take nourishment against their inclination, and to this end offer all kinds of dainties. We must not overlook this unnatural absurdity whenever the appetite fails, or aversion to food is present, or the digestive organs are affected by the disease, and are not in a condition to perform their functional assimilative process, or, as it is now called, metamorphosis of tissue. It is easy to comprehend that the nouishment imposed will not benefit the body, and that it is much more probable that the existing disease will receive an undesirable reinforcement, as the digestive organs in such a condition are made still worse than before. But it must be the most deleterious when the naturally depressed appetite is sought to be improved by palliative irritants. Food and drinks are administered which frequently contain articles having medicinal virtues, which are extremely seldom suitable for the case, and even if they should happen to be so in an exceptional instance the doses are so large and unsuitable as to be not less hurtful. It is, therefore, always and unexceptionally true *that every nourishment not desired by the patient, let it be what it may, can only be given to his injury* if the disease is a fever or anything else. In all cases nature speaks so clearly and definitely that every mistake is impossible if one only gives to her language the necessary attention, and one does not commit the culpable usurpation of assuming to be her master instead of her minister.

I hope I may be pardoned the presumption of adding anything to what is said by this, the ablest disciple of Hahnemann, but it is only in the same line of thought Bœnninghausen here says what we must not do in feeding the patient. I will only attempt to show the obverse, and extend to other cases than nourishing the sick. What may we permit them to eat and drink? Hahnemann says, *Organon*, Section 262: In acute diseases, on the contrary (insanity excepted), the fine, unerring inner sense of the active instinct of self-preservation will decide the course to be pursued so clearly that the physician will only have to advise the friends and attendants to obey this voice of nature by gratifying the patient's ardent desires, without offering and urging to accept hurtful things.

Section 263. The food and drink most commonly craved by patients suffering from acute diseases is generally of a palliative and soothing kind, and not properly of a medicinal nature, but merely adapted to the gratification of a certain longing. Slight obstacles which moderate gratification might place in the way of recovery are more than counterbalanced by the power of a homœopathic medicine, by the vital force liberated by the medicine, and by the refreshing effect of a gratified desire. In acute diseases the temperature of the chamber and the quantity of covering should be regulated entirely according to the wishes of the patient; while every kind of mental exertion and emotional disturbance is to be carefully avoided.

Hahnemann's views on the diet in chronic diseases are contained in Section 261. The proper regimen to be enjoined during the use of medicines in chronic diseases consists in the removal of all obstacles in the way of recovery, and in the substitution of a wholesome mode of life, such as innocent recreation of the mind, active exercise in the open air in all kinds of weather (daily walks, light manual labor), proper, nutritious food, and drink unadulterated with medicinal substances.

Hahnemann and Bœnninghausen leave us in doubt as to what we are to do where patients have formed habits of using injurious agents, drinking liquors, using tobacco, etc. In acute diseases nature usually relieves us of this embarrassment, as we seldom find the morbid appetite active. But in chronic diseases the drunkard wants his dram and the smoker his pipe. In these cases we must consult the unperverted taste. To the novice alcohol in all its forms, unless disguised skillfully, is repugnant; tobacco makes him deathly sick; vinegar, pepper, spices, condiments are disagreeable, and only by persistence become endurable, and finally almost indispensable. If these things thus acted at first they are hurtful always, and should be discarded. But the case becomes complicated when they have become almost a second nature—for instance, the man who has used tobacco a third or half a century. Is the benefit to be obtained worth the struggle? In many cases it is not. We must use our judgment, unbiased by fanaticism on the one hand, nor must we yield too indulgently on the other. And a habit that, in a hygienic sense, is worse than the tobacco habit, is coffee-drinking. Many a case of neurasthenia and the many ills of nervous women are caused by coffee. And two or three months' abstinence, without medicine or change of habits even, will restore the patient to health and usefulness.

